# Oxytocin Amplifies Negative Response to Ambiguity in Adolescent Females With and Without Eating Disorders

**DOI:** 10.1002/erv.3167

**Published:** 2025-01-30

**Authors:** Victoria Burmester, Emerie Sheridan, Nikita Catalina Julius, Jordan Elliott, Olivia Thackeray, Dasha Nicholls

**Affiliations:** ^1^ Division of Psychiatry Faculty of Medicine Hammersmith Hospital Imperial College London London UK

**Keywords:** anorexia nervosa, bulimia nervosa, development, neuropsychology, treatment

## Abstract

**Objective:**

Eating disorders (ED) typically emerge in adolescence, a critical period for brain development and peer bonding. Interpersonal difficulties—particularly social anxiety—frequently co‐occur with ED. Oxytocin is a neuropeptide that modulates social cognition and linked to prosocial effects. To date, no study has investigated oxytocin's effects on negative interpretation bias toward ambiguous information in adolescents with ED.

**Methods:**

Forty‐eight female adolescents aged 16 to 17 years with and without EDs took part in a placebo‐controlled, double‐blinded, randomised, crossover trial investigating the effects of 24 IU intranasal oxytocin on negative interpretations of ambiguous scenarios. Participants and controls were tested twice, approximately one week apart.

**Results:**

Contrary to hypothesis, oxytocin increased negative interpretations overall (*p* = 0.019, large effect). Adolescent females with AN or BN made more negative interpretations than controls when presented with ambiguous information. There was no group effect for those who reached or did not reach threshold on an autism screen.

**Conclusions:**

This study suggests adolescents with EDs interpret ambiguous information more negatively than controls and that oxytocin administration amplifies negative responses to ambiguity in adolescent females, including in controls. Research tools that effectively identify these biases would help to widen the scope of ED treatments for adolescents.

**Summary**

- Adolescent girls with an eating disorder interpret ambiguity more negatively than controls.
- Oxytocin may increase a social fear signal in adolescents when interacting with uncertainty.
- The results indicate that oxytocin administration may not benefit adolescent girls.

## 1 Introduction and Aims

Anorexia nervosa (AN) and bulimia nervosa (BN)—the most studied eating disorders (ED)—typically emerge in adolescence, a transitional developmental period between puberty and brain maturation in mid‐20s, where profound social and neurological change takes place (Blakemore 2012). During adolescence, peer interactions are of increased importance and individuals exhibit enhanced responsiveness to social stimuli (Orben, Tomova, and Blakemore 2020) and a heightened desire for social approval (Blakemore 2018). The incidence of body dissatisfaction and disordered eating is strongly associated with societal overvaluation of appearances and the thin ideal (Aparicio‐Martinez et al. 2019; So et al. 2023).

Individuals with ED exhibit impaired social functioning, which is an individual's ability to interact with their social environment effectively (Patel, Tchanturia, and Harrison 2016). The enforced social isolation during the COVID‐19 pandemic posed a challenge to normal adolescent social development and resulted in a dramatic increase in adolescent ED prevalence (J Devoe et al. 2023). In young adult females with AN or BN, the degree of negative interpretation bias demonstrated in ambiguous social contexts positively correlates with clinical symptoms (Cardi et al. 2017; Burmester et al. 2023). These findings were replicated in adolescents with AN or BN, who demonstrated greater negative interpretation bias in socially ambiguous sentence completion tasks than controls (Rowlands et al. 2021). In addition to maintaining EDs, in individuals with AN or BN, premorbid fear of negative evaluation predicts ED symptoms (Cardi, Tchanturia, and Treasure 2018). Conversely, social improvements may prevent onset of ED and facilitate successful recovery (Stice et al. 2017). Autism is both a risk factor for ED development and a common comorbidity (Saure, Laasonen, and Raevuori 2021; Solmi et al. 2021); adolescents with ED frequently present with autistic traits, which are not routinely addressed in treatment unless serious.

Oxytocin is a nonapeptide implicated in successful social functioning and widely expressed in mammalian brains with a receptor distribution that varies by species, sex, and individual factors (Ross et al. 2009). Oxytocin's social effects are demonstrated both preclinically and in clinical cohorts, and linked to social reward via serotonergic systems, although with mixed findings (Dölen et al. 2013). Two meta‐analytic reviews examining exogenous oxytocin and social function across diverse clinical and control groups concluded that oxytocin had small or null effects on social functioning in clinical groups and small prosocial effects in control populations (Bakermans‐Kranenburg et al. 2013; Ooi et al. 2017). However, both meta‐analyses pooled heterogeneous social tasks and single versus repeated doses across differing clinical groups. Since oxytocin's effects are highly dependent on context and person, the validity of such heterogeneous meta‐analyses might be compromised (Bartz et al. 2011).

The aim of this study was to determine whether adolescent females with ED demonstrate a greater negative interpretation bias in response to ambiguous scenarios compared to female adolescents without an ED, and whether intranasal oxytocin administration, or scores on a threshold‐screen for autism would impact interpretation of ambiguous information. It was hypothesized that 24 IU of intranasal oxytocin would reduce negative interpretations overall and that participants with an ED or meeting threshold for ASD would exhibit a greater negative response to ambiguous social stimuli than controls.

## 2 Methods

### 2.1 Design

**FIGURE 1:**
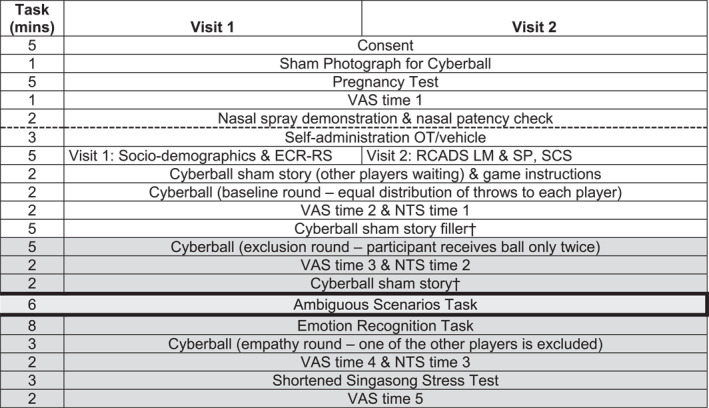
Timeline of sessions in parent study. † Sham time filler: participants instructed to continue with bespoke leisure activities questionnaires whilst waiting for other players. ECR‐RS = experiences in close relationships—Relationship structures; NTS = needs threat scale (cyberball); OT = oxytocin; RCADS = revised children's anxiety and depression scales (LM = low mood; SP = social phobia); social connectedness scale; VAS = visual analogue scale. Gray shading indicates measures completed during the hypothesized window of oxytocin's effects. ECR‐RS = Experiences in Close Relationships—Relationship Structures; NTS = Needs Threat Scale; OT = oxytocin; RCADS = Revised Child Anxiety and Depression Scale (LM = Low Mood; SP = social phobia); SCS = Social Connectedness Scale; VAS = visual analogue scales.

This study was part of a randomised controlled trial (RCT) investigating a range of social functions in adolescent females with and without AN or BN (see Figure 1). The trial design was placebo‐controlled, double‐blinded, randomised, and participants were tested twice, approximately one week apart.

### 2.2 Participants

**FIGURE 2:**
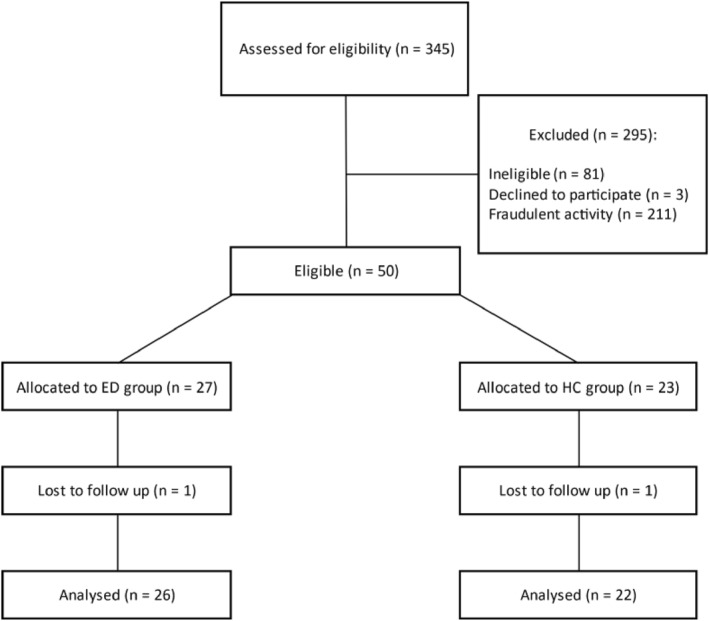
Participant recruitment through the study. Fraudulent applications from autonomous programmes (bots) were identified using Expert Review Fraud Detection on Qualtrics.

Forty‐eight adolescent females aged 16 to 17 years with and without an ED were recruited using social media advertisements (Figure 2). ED participants confirmed being in treatment for their ED and provided their NHS number. All participants had to be able to travel to the Children's Clinical Research Facility at St. Mary's Hospital, London. Exclusion criteria for both groups were co‐morbid obsessive‐compulsive disorder, bipolar disorder, schizophrenia, or history of psychosis; insufficient English language skills; uncorrected hearing or sight impairment; regular or accomplished solo singers because the stress‐inducing task involved singing; pregnancy or breastfeeding; and recent experience of a significant life event, such as bereavement, which alters normal mood states and impacts performance. It was noted whether participants took an oral contraceptive pill due to the potential influence of menstrual cycle on endogenous oxytocin concentrations.

### 2.3 Measures & Materials

COVID‐complaint personal‐protective equipment was provided by the hospital. The placebo (vehicle) and 24 international units (IU) of Syntocinon intranasal sprays were supplied by Victoria Apotheke, Switzerland. Urine pregnancy tests determined pregnancy status, data were collected on computer tablets and mirrors were used for the Glatzel test.

A sociodemographic questionnaire collected information on gender identification, age, ethnicity, type of social support accompanying them (i.e. parent, friend, sibling, or romantic partner), time since last period, time since most recent meal, handedness, caffeine consumption, parental education level and hours slept previous night. Visual analogue scales (VAS) measuring 100 mm measured feelings of importance, confidence, anger, and stress, anchored by ‘not at all’ and ‘as much as I can imagine’.

The parent trial investigated oxytocin's effects on other social measures not analysed here. These included the social ostracism game, Cyberball (Van Beest et al. 2006); information on leisure activities, Emotions Recognitions Test (www.diagnoseis.com) and the Shortened Sing‐a‐Song Stress Test (Burmester et al. 2019).

Eating disorder psychopathology was measured using the 22‐item Eating Disorders Diagnostic Scale (EDDS) that measures whether self‐reported ED psychopathology reaches ED diagnostic thresholds in individuals between the ages of 13 and 65 years (Stice, Telch, and Rizvi 2000). The present study included only individuals reaching clinical threshold. We used the condensed 10‐item version of the 50‐item Autism‐Spectrum Quotient, validated in 16‐17‐year‐olds (Baron‐Cohen et al. 2001), to assess autistic traits. For each statement, participants select one of four responses: slightly or definitely agree or disagree. A score above 5 out of 10 suggests an autism spectrum condition.

The 20‐item Ambiguous Scenarios Test for Depression in Adolescents (Orchard, Pass, and Reynolds 2016), which measures depressive interpretation bias, was updated to contemporary social norms: for example, ‘email’ replaced ‘letter’. Ambiguous scenarios were presented to participants, who were instructed to provide a short, written ending for each scenario (up to five words). Answers were rated by two independent researchers into three categories: (1) negative, (2) positive and (3) mixed or a neutral interpretation without emotional valence. There were only two disagreements, which were resolved by a third rater. The interpretations were averaged for each testing session.

To measure social anxiety and depression, the social phobia (SP) and low mood (LM) subscales of the Revised Child Anxiety and Depression Scale (RCADS) were used (Chorpita, Ebesutani, and Spence 2015). The potential influence of the social support's presence was described and explored with the 9‐item Experiences in Close Relationships – Relationship Structures (ECR‐RS) (Fraley et al. 2011), designed to assess attachment patterns in a variety of relationships, including parents, friends, and romantic partners and validated in 16‐17‐year‐olds.

### 2.4 Procedures

The Imperial College sponsored study (22/EM/0044; IRAS project ID: 297695) ran from July 1 to October 14, 2022. Participants who met study criteria provided informed consent and completed an online registration survey, which asked about socio‐demographics, puberty data, and included the EDDS. On the day, informed consent was provided again, signed digitally, at the start of visit 1. Researchers wore trial‐logoed t‐shirts and, to comply with COVID‐19 safety measures, wore face masks in clinical hospital areas, including the testing room, which comprised a hospital bed (not used), tables, chairs and an ensuite toilet. Social‐distancing restrictions were still in place in the Children's Clinical Research Facility, so waiting areas were not available; however, one visitor was permitted to enter the testing room. Since it was not deemed reasonable to require 16‐ and 17‐year‐olds to attend alone during the pandemic and because there is evidence that the presence of a social support might potentiate oxytocin's effects (Heinrichs et al. 2003), we instead required the presence of one visitor (social support person) and assessed the closeness and style of the relationship to explore potential confounding effects (Chorpita, Ebesutani, and Spence 2015).

Each visit lasted approximately one hour, and the same social support person was present for each visit. At the end of the second and final visit, a ‘manipulation check’ asked whether patients thought they had received oxytocin or placebo in that session. It was noted if participants had consumed caffeine or alcohol in the last 12 h, whether they smoked or vaped, and if they had allergies or conditions that day causing nasal congestion that day. To comply with ethics, participants took a pregnancy test and confirmed a negative result. To identify nasal patency, under researcher supervision, participants cleared their nostrils, then blew out and inhaled several times through each nostril whilst blocking the other, to decide which nostril air passed through easiest or quickest; this was confirmed using the Glatzel mirror test. Participants next self‐administered either placebo or 24IU oxytocin with their head tilted back via one initial long inhalation followed by sniffing for 30 s to prevent the drug exiting. There were six actuations every 30 s. The drug was delivered to either the dominant or nondominant nostril; this was determined a priori using a pseudo‐randomised alternating design. We estimated the window of oxytocin's social effects to occur from 20 to 90 min following intranasal administration (D. Martins et al. 2020; Gossen et al. 2012; Turton et al. 2018; Munesue et al. 2021) (Figure 1). Each session lasted about an hour. Participants and social supports were compensated with a £50 and £20 online voucher, respectively.

### 2.5 Data

Data were analysed using Statistical Package for the Social Sciences (SPSS) version 29. The ambiguous scenarios measure was randomly divided into two parts for sessions 1 and 2 (10 questions each), counterbalanced, and each session's mean score calculated.

To investigate the effects of oxytocin and group on negative interpretations, a repeated‐measures analyses of variance (RMANOVA) was performed with ED group and autism traits as between‐subject variables. In exploratory analyses, menstrual cycle phase, hours of sleep the previous night, and postprandial time were incorporated as covariates to explore potential drug interaction effects.

## 3 Results

### 3.1 Participant Characteristics

Twenty‐six females with high ED psychopathology (restricting = 23, binge‐purge = 5) and 22 controls were recruited (Table 1) with BMI from 15.61 to 35.04 kg/m^2^ (M [SD] = 22.24 (5.12) kg/m^2^) (see Table 2). Three participants reported using their left hand to write (6.3%), all female‐at‐birth participants identified as female gender. The sample was not able to identify whether they were given oxytocin or placebo (*p* = 0.423) in the final visit manipulation check.

**TABLE 1:** Participant socio‐demographics.

	Total	ED group, *n* = 26	Controls, *n* = 22
Ethnicity, *n* (%)			
White	16 (33.3)	8 (30.8)	8 (36.3)
Black	12 (25.0)	8 (30.8)	4 (18.2)
Asian	11 (22.9)	7 (23)	4 (18.2)
Mixed	7 (14.6)	3 (11.4)	4 (18.2)
Other	2 (4.2)	—	2 (9.1)
Social support relationship, *n* (%)			
Friend	32 (66.7)	19 (73)	13 (59.9)
Parent/guardian	8 (16.7)	2 (7.7)	6 (27.3)
Sibling	4 (8.3)	2 (7.7)	2 (9.1)
Romantic partner	3 (6.3)	2 (7.7)	1 (0.1)
Other	1 (2.1)	1 (3.8)	—
Parental education, *n* (%)			
At least one with university degree	26 (54.2)	15 (57.7)	11 (50)
No university degree	19 (39.6)	9 (34.5)	10 (45.5)
Unsure	3 (6.3)	2 (7.7)	1 (4.5)
			(*n* = 48)

**TABLE 2:** Participant characteristics.

	Mean (SD)		ED versus control independent *t* test (*p* value)	Norm mean	Control versus norm (*p* value^a^)	Cronbach's *α*	McDonald's *ω*
	ED (*n* = 26)	Control (*n* = 22)					
RCADS^b^	19.62 (5.30)	13.68 (4.01)	< 0.001**	12.85	0.456	0.861	0.857
social phobia							
RCADS^b^	14.77 (5.66)	8.45 (3.70)	< 0.001**	9.36	0.767	0.878	0.874
low mood							
AQ‐10^c^	4.88 (2.28)	3.45 (1.60)	0.018*	2.77	0.1142	0.603	0.563
ECR‐RS^d^							
Avoidance	2.70 (1.34)	2.42 (0.74)	0.359	3.18	< 0.001**	0.849	0.851
Anxiety	3.30 (1.48)	2.06 (1.20)	0.004*	2.53	0.064	0.810	0.841

*Note:* Normative mean ages 16.3. Normative means (Fraley et al. 2011); Revised Child Anxiety and Depression Scale (RCADS) baseline social phobia and low mood normative means, females aged 16–18 years (Chorpita, Ebesutani, and Spence 2015).

Abbreviations: AQ‐10 = Autism Spectrum Quotient 10; ED = eating disorder; Experiences in Close Relationships—Relationship Structures (ECR‐RS); SD = standard deviation.

^a^ one‐sample *t* test.

^b^ (Mathyssek et al. 2013); 13.52.

^c^ (Allison, Auyeung, and Baron‐Cohen 2012); 16.44 years.

^d^ (Feddern Donbaek et al. 2014).

* = *p* < 0.05, **p < 0.001 (independent *t* tests compared ED & control groups samples).

### 3.2 Negative Interpretations

**FIGURE 3:**
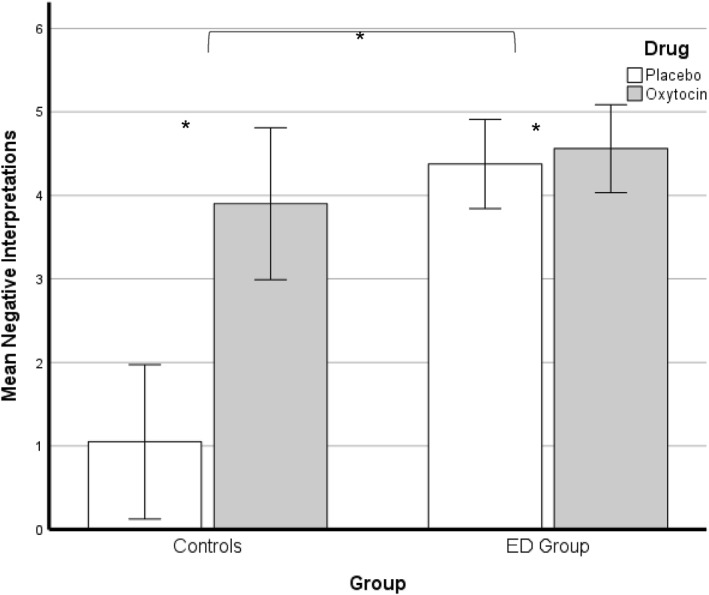
Frequency of Negative Interpretations in Control and Eating Disorder Group after placebo and oxytocin.

Oxytocin administration (*M* = 3.44, SD = 1.38) increased negative interpretations of ambiguous social scenarios with a large effect across the sample of adolescents compared to placebo (*M* = 3.06, SD = 1.37): F (1,43) = 5.97, *p* = 0.019, partial *η* ^2^ = 0.12. There was a main effect of belonging to the ED group (F [1,43] = 5.38, *p* = 0.025, partial *η* ^2^ = 0.111), with the ED group making more negative interpretations than controls (Figure 3). There was no effect on negative interpretations in those reaching threshold autism (F [1,43] = 1.18, *p* = 0.283) on negative interpretation scores.

Interactions: There was a medium‐effect interaction between group and drug (F [1,43] = 4.61, *p* = 0.037, partial *η* ^2^ = 0.097), such that oxytocin increased negative interpretations in controls to a greater degree [F (1,43) = 5.38, *p* = 0.037, partial *η* ^2^ = 0.097]. There was a three‐way interaction of oxytocin administration, ED group and autism traits with a large effect, F(1,43) = 6.17, *p* = 0.017, partial *η* ^2^ = 0.13.

### 3.3 Exploratory Analyses

#### 3.3.1 Positive & Neutral Interpretations

There was no main effect of oxytocin administration the number of positive interpretations made compared to placebo: F (1,43) = 0.30, *p* = 0.59. ED status did not have a significant main effect on the number of positive interpretations made (F [1,43] = 3.47, *p* = 0.069). Oxytocin's effects did not interact with ED status (F [1,43] = 1.53, *p* = 0.224) or autism traits (F [1,43] = 0.51, *p* = 0.822). There were too few neutral responses for parametric analysis; on average, the neutral interpretation frequency was less than one (0.48).

#### 3.3.2 Potential Confounders

At baseline, pre‐drug administration, the ED group reported higher scores of stress with a large effect (t [2.98] = 2.23, *p* = 0.002, *d* = 0.86). Menstrual cycle phase did not have interact with oxytocin on the interpretation of ambiguous social scenarios (all *p*‐values > 0.106). Hours of sleep the previous night (*M* = 7.01, SD = 1.63) and postprandial duration (*M* = 5.17, SD = 5.29 h) did not interact with oxytocin (all *p*‐values > 0.074).

## 4 Discussion

To our knowledge, our study is the first to examine intranasal oxytocin's effects on adolescent females with and without an ED—both restricting and binge‐purge type—and to investigate its effects on interpretation of ambiguous information. Administration of 24 IU of oxytocin increased the number of negative interpretations of ambiguous information but was incommensurate across groups. Interaction effects implicate autism traits as a potentially important factor in interpretation biases toward ambiguous information.

Despite systematic reviews attempting to explain contradictory oxytocin findings using social boundary theory, which suggests that prosocial behaviour arises in situations perceived as ‘safe’ and defencive behaviour follows from circumstances viewed as ‘unsafe’, no compelling theoretical framework explains oxytocin's lack of effects in clinical groups. Our finding that oxytocin increased a negative response to social ambiguity adds to a growing literature documenting oxytocin's negative social effects. This lends weight to the theory that the anxiolytic effects of oxytocin might be context‐dependent (Evans et al. 2014; Amico et al. 2004) and rather than oxytocin augmenting the salience of a ‘connectedness’ signal (The Social Salience Hypothesis of Oxytocin, SSH) (Shamay‐Tsoory et al. 2016), oxytocin may augment a social fear signal amid uncertainty (Watts et al. 2022). In AN populations intolerance of uncertainty is commonly seen, whereby uncertainty generates stress, and is related to a perceived need for control (Brown et al. 2017). Together with a hypocaloric state, potential stress from ambiguity, may have contributed to the low mood of those in the ED group.

The increase of negative bias when interpreting social information was observed despite a high proportion of controls achieving subthreshold or high ED symptom counts, suggesting a potential role for duration of illness and concomitant psychopathology in low scores on social measures. This is an important finding as it indicates that deteriorating social competence might be a risk factor for disease progression from sub‐clinical to full‐blown ED status and a factor that reinforces the importance of prevention and early intervention in ED.

Our findings suggest that ambiguous information presented to adolescent populations may elicit negative responses, exacerbated by oxytocin. Further, ED populations are more likely to have a negative interpretation bias than those without an ED, and oxytocin might interact with a potential cortisol response in individuals who find ambiguity stressful. There were very few neutral interpretations in this cohort, which may reflect cognitive rigidity associated with chronic negative‐energy imbalance or burgeoning mentalization and self‐assuredness in this age group (Robertson et al. 2021; Crone et al. 2020).

The emergence of a negative bias in adolescent girls with ED and a disadvantageous interaction response to oxytocin in those who also met the threshold for suspected autism, suggests that interpersonal difficulties present in mid‐adolescence are potentially important transdiagnostic factors, see the Central to the Transdiagnostic Model of ED (Fairburn, Cooper, and Shafran 2003), during a developmental stage of identity development (Wittek et al. 2021). However, although social difficulties in ED and autism could appear homogenous with respect to deficits in differentiation ability, perhaps the shared negative response may only become significant in the context of ED or, indeed, any other mental health comorbidity that might result in increased stress response to ambiguity.

**FIGURE 4:**
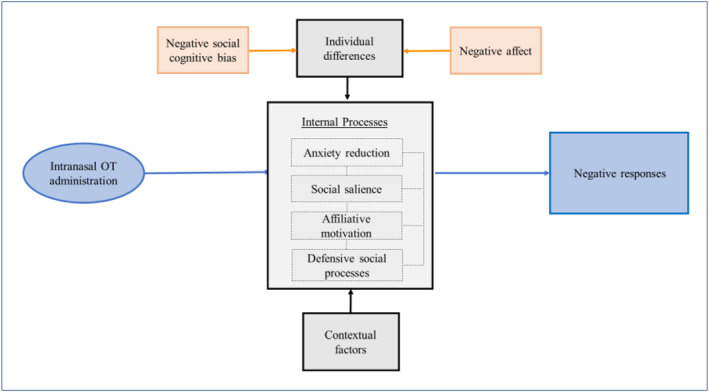
Interactionist Model of Negative Effects of Intranasal Oxytocin in female adolescents. Model adapted from Bartz et al. (Bartz et al. 2011). Moderators in orange are necessary components to produce a negative social response to oxytocin. Contextual moderators can include environmental and social stimuli.

The SSH proposes that an oxytocin response is moderated by contextual and individual characteristics (Shamay‐Tsoory et al. 2016) (Olff et al. 2013). However, common neurocognitive processes seen in ED and autism—and associated psychopathology—might override environmental factors. Since oxytocin is not associated with mood lowering (IsHak, Kahloon, and Fakhry 2011), the negative affect and subsequent differential and disadvantageous response to oxytocin might derive from the interplay of multiple negative factors such as negative expectations arising from low mood or, in some cases, malnourishment. Thus, we propose the conditions of negative affect and negative interpretation bias are necessary and sufficient to explain the negative oxytocin response to ambiguity in this sample (Figure 4).

Study limitations were the absence of a comparison group using participants with no social support. Whilst most participants were accompanied by a friend; a small minority brought a family member or romantic partner. Although different social‐support relationships may have impacted endogenous oxytocin concentrations, we explored the closeness of relationships and found no effect. The study considered the potential confounders of menstrual cycle and sleep. Menstrual cycle did not influence results. Sleep deprivation increases plasma oxytocin concentrations in females (Schuh‐Hofer et al. 2018) and endogenous oxytocin is both circadian—higher at night in humans—and released by eating (Uvnas‐Moberg et al. 2005). Here, both factors were found not to influence outcomes.

This study was conducted during the COVID‐19 pandemic with widespread social restrictions and stay‐at‐home orders in place. EDs increased exponentially during the pandemic, and social isolation may relate to negative baseline scores of importance and confidence in those with high levels of ED psychopathology than controls. Additionally, the SARS‐COV2 virus can induce neurological changes to olfactory brain regions; Anosmic individuals were, therefore, excluded to minimize this (Douaud et al. 2022). However, Douaud et al. showed that neurological changes also occurred in asymptomatic COVID‐19 individuals (Douaud et al. 2022). Given the route of drug administration, this might have led to differences in the quantities of oxytocin reaching the brain.

EDs are frequently co‐morbid with autism and with social anxiety disorder. This work needs replicating in larger clinical populations with and without these comorbidities. Given oestrogen's promotion of central oxytocin (Ochedalski et al. 2007), this work does not generalize to males. Route of administration and dose‐response effects need exploring, as evidence of stronger effects at lower oxytocin doses than used here should be examined (D. Martins et al. 2021). These findings may also benefit from replication using the TTA‐121 form of synthetic oxytocin that has enhanced bioavailability (Inoue et al. 2010).

## Conflicts of Interest

The authors declare no conflicts of interest.

## Data Availability

The data that support the findings of this study are available on request from the corresponding author. The data are not publicly available due to privacy or ethical restrictions.
